# 

**Published:** 1904-06-15

**Authors:** 


### CONTENTS.

A Comparison between the Jenkins Porcelain Enamel and the High-fusing Porcelain, *By J. Q. Byram, D.D.S.* 271

Management of the Inlay Matrix, *By L. E. Custer, A.M., D.D.S.* 282

The Province of Porcelain Work in Dentistry, *By Hart J. Goslee, D.D.S.* 285

Editorial, - - - - - - - - - - - 313

Announcements, - - - - - - - - 314

Obituary, - - - - - - - - - - - 320

Indents, - - - - - - - - - - - 323

